# Epithelial-Mesenchymal Transition and Breast Cancer

**DOI:** 10.3390/jcm5020013

**Published:** 2016-01-26

**Authors:** Yanyuan Wu, Marianna Sarkissyan, Jaydutt V. Vadgama

**Affiliations:** 1 Division of Cancer Research and Training, Department of Medicine, Charles R. Drew University of Medicine and Science, Los Angeles, CA 90059, USA; mariannasarkissyan@cdrewu.edu (M.S.); jayvadgama@cdrewu.edu (J.V.); 2 Jonsson Comprehensive Cancer Center, David Geffen School of Medicine, University of California at Los Angeles, Los Angeles, CA 90095, USA

**Keywords:** breast cancer, epithelial cells, metastasis

## Abstract

Breast cancer is the most common cancer in women and distant site metastasis is the main cause of death in breast cancer patients. There is increasing evidence supporting the role of epithelial-mesenchymal transition (EMT) in tumor cell progression, invasion, and metastasis. During the process of EMT, epithelial cancer cells acquire molecular alternations that facilitate the loss of epithelial features and gain of mesenchymal phenotype. Such transformation promotes cancer cell migration and invasion. Moreover, emerging evidence suggests that EMT is associated with the increased enrichment of cancer stem-like cells (CSCs) and these CSCs display mesenchymal characteristics that are resistant to chemotherapy and target therapy. However, the clinical relevance of EMT in human cancer is still under debate. This review will provide an overview of current evidence of EMT from studies using clinical human breast cancer tissues and its associated challenges.

## 1. Introduction

Breast cancer is the most common cancer in women and ranks second among cancer deaths in women. Developing metastasis is the main cause of death in breast cancer patients. Based on gene expression profiling, breast cancer has been characterized into Luminal A, Luminal B, HER2 (human epidermal growth factor receptor 2)-enriched, and basal-like subtypes—each of which has been shown to have different prognoses [1,2,3,4]. Luminal A, Luminal B and HER2-enriched tumors retain most epithelial features, while basal-like tumors exhibit both basal and mesenchymal features [5,6]. Basal-like breast cancer cells are constitutively more invasive. In addition, HER2-enriched tumors are also more likely to develop metastatic disease. Even though the anti-HER2 antibody (Herceptin, also known as trastuzumab) treatment has been successfully used to treat metastatic HER2 breast cancer, the *de novo* and/or acquired resistance is still a major issue with trastuzumab treatment [7,8,9]. A high proportion of HER2-overexpressing breast cancer patients who experience resistance to trastuzumab progress to develop brain metastases. The two-year survival rate for brain metastasis is less than 2% [8,9].

To date, the epithelial-mesenchymal transition (EMT) phenomenon has been the favored explanation of distant metastases for epithelial cancers including breast cancer. The essential features of EMT are associated with disruption of intracellular tight junctions and loss of cell-cell contact. This results in the loss of epithelial features and the gain of mesenchymal morphology. These cells exhibit an increase in cell self-renewal and an increase in heterogeneous subpopulations. These features enhance cell motility, resulting in the release of cells from the parental epithelial tissue site. These cells gain the ability to reconstitute metastatic colonies at distant sites [10,11]. The prevailing model of metastasis suggests that a small subpopulation of cancer cells acquires cancer stem-like cell (CSCs) traits [12], exhibits mesenchymal cell characteristics, and migrates away from the primary tumor site and progresses to distant metastatic sites [13]. Tumor cells which have undergone EMT also display features similar to experimentally defined CSCs, such as similar cell surface markers to those of CSCs and reduced cell proliferation rates [14,15].

However, to date, EMT is largely demonstrated within the theoretical framework built on observations from *in vitro* cell culture models. The pathologic evidence of EMT in human cancer tissue samples has not yet been well established. Demonstrating EMT in human cancer is complicated by the fact that most tissue samples obtained from human tumors were taken at various time points—either from primary or metastatic sites. The tissues from these sites may have either not yet undergone EMT or undergone the reverse process of mesenchymal-to-epithelial transition (MET) [16,17,18,19], respectively. Histopathological analysis can expediently differentiate between epithelial cancer cells and fibroblasts based on the prototypical spindle-shaped morphology of the fibroblasts. However, epithelial cells which have undergone EMT and have converted to the mesenchymal type of cells are hardly distinguishable from fibroblasts. Therefore, it has been a challenge to assess tumor progression based on “EMT” in current clinical practice, especially through traditional pathological assessment.

The challenges from traditional assessment methods can be partially overcome by utilizing gene profiling. It has been established that multiple molecular changes are required for epithelial cells to gain a mesenchymal phenotype. Studies from gene profiling show that numerous genes are differentially expressed during the EMT process [20,21,22]. Several interrelated pathways and a cluster of signaling molecules have been identified to be involved in the EMT process as well as in the tumor progression and metastases that occur subsequently [20,21]. These signals could play key roles in governing the cancer cells through the EMT program, thereby predicting clinical outcomes of cancer patients.

Here, we will focus on discussing characteristics of EMT that may be evaluated in clinical samples for accessing EMT initiation and determining tumor progression in breast cancer.

## 2. EMT and Its Plasticity Features

**Figure 1:**
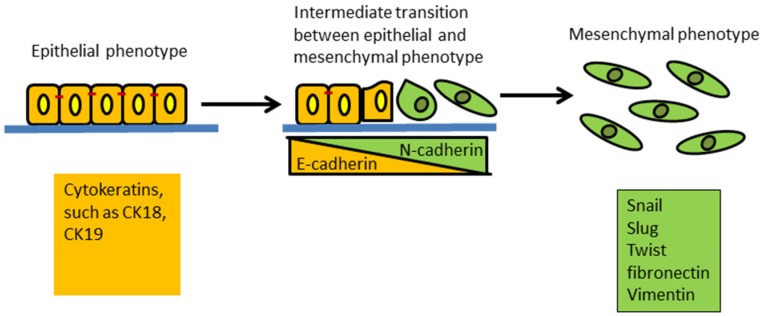
Schematic of the epithelial to mesenchymal transition (EMT). This process results in a transformation and transition of polarized epithelial cells toward mobile mesenchymal cells. Expression of cytokeratins is a feature of epithelial cells. The increased expression of Snail, Slug, Twist, fibronectin and Vimentin is common in mesenchymal cells. The E-cadherin to N-cadherin switch indicates epithelial cells are progressing through EMT.

Breast cancer originates from epithelial tissue and, hence, originally is characterized by a typical “sheet-like” morphology with apical-basal polarity, with intact tight and adherent junctions, which are separated from the underlying tissues by the basement membrane (a thin layer of specialized extracellular matrix). Mesenchymal cells are oppositely characterized by loosely associated cells and disorganized cellular layers that lack polarity and tight cell-to-cell adhesion proteins. The morphology of mesenchymal cells is better adapted to cell migration. EMT is typically characterized as loss of epithelial cell adhesion protein E-cadherin and cytokeratins together with the gain of mesenchymal-associated molecules N-cadherin, Vimentin, and fibronectin (Figure 1). The process is described as “cadherin switching”, *i.e.*, down-regulation of E-cadherin and up-regulation of *N*-cadherin [23,24]. The status of these biomarkers has not been fully evaluated in clinical breast cancer tissues undergoing EMT. However, the expressions of some EMT-associated biomarkers have been detected in a variety of clinical human cancer tissues including breast cancer. Several studies examined EMT-related markers, such as Vimentin, N-cadherin, Snail, Slug, Twist, N-cadherin and cytokeratins expression, in different subtypes of breast cancer tissues by immunohistochemistry (IHC). The data suggest that the EMT-related markers were more likely to be expressed in the basal-like subtype of breast cancer and are related to the aggressiveness of the tumors [12,25,26,27,28]. Hence, the expression of EMT markers may be used to infer EMT-like changes in breast cancer tissues.

The cadherin switch (*i.e.*, E-cadherin^−^/N-cadherin^+^ phenotype) is found to be more frequently observed in HER2+ receptor subtypes of breast cancer, as described by Aleskandarany *et al.* [27]. However, a study conducted by Tsang *et al.* failed to show a predictive role of EMT marker expression in disease outcomes [29]. In their study, expression of P-cadherin and Vimentin were examined by IHC in a large cohort of breast carcinomas (*n* = 1145). Their data suggested that P-cadherin and Vimentin could be adjunctive to the commonly used IHC surrogates for basal-like breast cancer identification, but not for predicting disease outcome.

In summary, it may be possible to assign some EMT markers to identify a distinct basal-like phenotype within a highly heterogeneous population of breast cancer cells. However, it remains to be determined if EMT is a mandatory process for metastasis to occur.

## 3. EMT and Breast Cancer Metastasis

It has been commonly believed that EMT may theoretically contribute to breast tumor metastasis. However, since the association of tumor metastases with EMT is mostly predicated on studies from cancer cell lines, the metastasis due to EMT in clinical scenarios needs to be validated. One of the primary challenges of conducting clinically relevant studies is that it is difficult to track individual cancer cells that have “transitioned or are transitioning” in ”real time” in human tissue. Typically, at the point of histopathological evaluation, the cell has either not yet undergone EMT, or has already undergone EMT or MET (mesenchymal-epithelial transition). Hence, the transition phase cannot easily be parsed and evaluated at a specific time or growth stage.

Recently Yu *et al.* established a dual-colorimetric RNA *in situ* hybridization (ISH) assay to characterize EMT in circulating tumor cells (CTCs) from breast cancer patients [30]. They examined seven pooled epithelial transcripts (Keratins 5, 7, 8, 18, and 19; EpCAM (epithelial cellular adhesion molecule) and E-cadherin) and three mesenchymal transcripts (fibronectin, N-cadherin, and SERPINE1/PAI1 (serpin peptidase inhibitor, clade E)) in 11 human breast cancer specimens. Rare primary tumor cells simultaneously express mesenchymal and epithelial markers in their study; however, mesenchymal markers were enriched in CTCs. Interestingly, they found that expression of the mesenchymal markers was more likely to be associated with clusters of CTCs rather than a single set of migratory cells. These results suggested that a single cell may be undergoing EMT into cluster cells, or a preexisting cluster of CTCs undergo mesenchymal transformation in the bloodstream. These data provide notable evidence of EMT occurring in relation to blood-borne dissemination of human breast cancer.

Evidence from additional studies suggests that it is conceivable to trace the EMT process by examining the dissociated cancer cells in circulation. Armstrong *et al.* have provided some clinical evidence of EMT in circulating tumor cells (CTCs) from patients with progressive metastatic solid breast and prostate tumors [31]. Armstrong and colleagues found co-expression of epithelial proteins such as EpCAM, Cytokeritins (CKs), and E-cadherin with mesenchymal proteins including Vimentin, N-cadherin, O-cadherin, and the stem cell marker CD133 in CTCs from 41 men with metastatic prostate cancer and 16 women with metastatic breast cancer. The CTCs from patients with advanced prostate and breast cancer displayed both epithelial and mesenchymal markers, suggesting that a switch or transition was taking place. Similar studies from Papadaki *et al.* and Kallergi *et al.* also detected EMT markers such as pan-cytokeratin, Twist, and Vimentin in CTCs from early and metastatic breast cancer patients [32,33]. Although specific markers for CTCs still require validation within a larger clinical setting, current evidence supports the hypothesis that EMT is involved in the metastatic process in human breast cancer. However, due to the biological heterogeneity of CTCs, the technical difficulty still remains in the detection and isolation of CTCs.

## 4. EMT and Cancer Stem-Like Cell (CSC)

Growing evidence from several studies suggests that tumor cells displaying cancer stem-like traits, through virtue of their stemness, could survive more easily from chemotherapeutic agents and targeted therapy. It is hypothesized that it is this subset of cells which subsequently becomes metastatic when oncogenic alterations occur.

**Figure 2:**
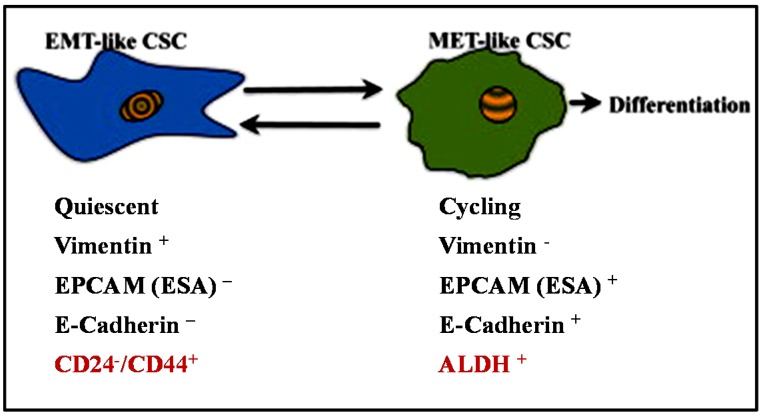
Breast CSCs display a cellular plasticity that allows them to transition between EMT and MET states. Hypothetical models show the characteristics of two different states of BCSCs as suggested by Liu *et al.* [36].

Phenotypically, breast cancer stem-like cells (BCSCs) express high CD44 (cell-surface glycoprotein CD44) and low CD24 (cell-surface glycoprotein CD24) (CD44high/CD24low) and also have increased ALDH1 (aldehyde dehydrogenase 1) activity (identified by ALDEFLUOR™ assay, StemCell Technologies) [18,34,35]. Liu *et al*. verified that distinct mesenchymal-like BCSCs are characterized by the CD44^high^ /CD24^low^ phenotype, and the epithelial-like BCSCs are characterized by the ALDH1^+^ phenotype (Figure 2) [36]. The mesenchymal-like BCSCs are primarily quiescent and usually localized at the tumor invasive margins whereas the epithelial-like BCSCs are proliferative and are located more centrally within the tumor. A significant study by Mani *et al.* fractionated stem-like CD44^high^/CD24^low^ cells by FACS from human mammary epithelial cells (HMLE) and identified that the mesenchymal-like BCSCs exhibit phenotypes similar to cells that have undergone an EMT [14]. The cells which displayed a CD44^high^/CD24^low^ phenotype had increased expression levels of Snail, Slug, Twist, N-cadherin and decreased E-cadherin. The cells introduced into monolayer culture also exhibited a mesenchymal morphology. The stem-like characteristics of the cells which had undergone an EMT were confirmed by *in vitro* spheres assay and *in vivo* mouse model. Clinical studies have shown that the CD44^high^/CD24^low^ phenotype was more prevalent in triple negative (ER/PR- and HER2-) breast cancer (TNBC) and is associated with increased risk for metastases to distant organs compared to other types of breast cancer [37,38,39,40,41].

## 5. EMT and Resistance to Treatment

The importance of EMT *in vivo* has been associated with resistance to chemotherapy in lung cancer recently [42]. Even though EMT was not required for lung metastasis in the mouse model in the study [42], therapeutic failure still could be a main cause of cancer metastases. Drug resistance has been correlated with the presence of the molecular “pump” in tumor cell membranes that actively expel chemotherapeutic drugs from the interior. This allows tumor cells to avoid the toxic effects of the drug or other molecular processes within the nucleus or the cytoplasm. The “pump” commonly found to confer resistance to chemotherapeutic agents in cancer is P-glycoprotein 1 (Pgp), also known as multidrug resistance protein 1 (MDR1). Increasing MDR1 expression was found to be associated with EMT processes in breast cancer cell resistance to the Adriamycin treatment [43]. Li *et al*. found that those MCF-7 breast cancer cells which become resistant to Adriamycin displayed enhanced invasion. Depletion of Twist1 expression in these cells blocked EMT and partially reversed drug resistance, suggesting that EMT contributes to resistance to Adriamycin [43]. Multiple studies have demonstrated that breast cancer cells’ resistance to paclitaxel/docetaxel and 5-fluorouracil is also accompanied by EMT [20,44,45,46]. Furthermore the EMT-associated resistant phenotype was reported for therapies targeting hormone and epidermal growth factor receptors as well. The ER-positive breast cancer cell line, MCF-7, became tamoxifen-resistant (MCF7-TamR) when it gained an EMT-like morphology—growing as loosely packed colonies with loss of cell-cell junctions and altered morphology [47]. The MCF7-TamR cells also expressed increased mRNA levels of Snail, Vimentin, and N-cadherin and decreased levels of E-cadherin, which are considered hallmark EMT characteristics [48]. Moreover, the EMT-like phenotype was also associated with HER2-overexpressing breast cancer cells resistant to trastuzumab [49,50]. A relative enrichment of EMT features was observed in the *de novo* resistance to trastuzumab in the HER2-positive breast cancer cell line JIMT1 [49] and acquired resistance to trastuzumab cell lines SKBR3/100-8 and BT474/100-2 that were generated from HER2-overexpressing breast cancer cell lines SKBR3 and BT474, respectively, in our laboratory [50].

Based on data from *in vitro* cell culture models, the crucial role of EMT in breast cancer resistance to chemotherapy and/or target therapies has been highlighted. However, we lack direct clinical evidence for EMT to cause drug resistance in solid breast tumors. Considering the postulated EMT model in tumor metastasis, epithelial tumor cells are likely to undergo EMT prior to entering circulation. A translational study was conducted to verify EMT in relation to chemotherapy using CTCs from breast cancer patients treated with neoadjuvant therapy [51]. In this study, Mego *et al.* investigated mRNA transcripts of EMT-inducing transcription factors (TFs) including Twist, Snail, Slug, ZEB1 and FOXC2 in tumor cells from the peripheral blood of 52 primary breast cancer patients by RT-qPCR. They defined the cut-off level of those transcripts using healthy donors’ samples and found that more than 15% of the samples overexpressed EMT-TFs [51]. Interestingly, over-expression of EMT-TFs was more likely to be detected in CTCs from patients who received neoadjuvant therapies. However, this study was not able to confirm the significance of neoadjuvant therapies in expressing EMT-TFs in CTCs due to the limitation of the study design and sample size. Nonetheless, the data provide links between EMT and treatment resistance in clinical samples. Additional studies are required to identify and/or validate markers for detecting cancer cells undergoing EMT in solid tumor or in CTCs.

## 6. Molecular Mechanisms of EMT

### 6.1. Signaling Pathways Mediate EMT

Understanding the molecular mechanisms responsible for EMT-mediated drug resistance and tumor metastasis will be essential for the discovery of new strategies to prevent EMT and restore the sensitivity of cancer cells to therapeutic treatment. EMT can be triggered by a diverse set of stimuli that includes growth factor signaling, cytokines, and tumor-stromal cell interaction. The transcriptional factors (TFs) Snail, Slug, Twist and zinc-figure E-box-binding homeobox (ZEB1 and ZEB2) are classified as EMT inducers. These TFs can induce EMT via different cell signaling pathways. To date, the TGF-β (Transforming Growth Factor β), Wnt (wingless-type MMTV (mouse mammary tumor virus))/β-catenin, and Notch (a family of transmembrane proteins) pathways have been strongly implicated in inducing EMT in epithelial cells including breast cancer epithelial cells.

#### 6.1.1. TGF-β Signaling

The most classical experimental model is the induction of EMT by TGF-β in epithelial cell culture. Upon TGF-β induction, the type II receptor (TGFR2) is activated and phosphorylates the type I receptor (TGFR1), thereby activating the Smad pathway and inducing EMT [52,53,54]. In the Smad-dependent pathway, TGF-β activates the type II receptor (TGFβRII), resulting in phosphorylation of the type I receptor (TGFβRI), and then activation of Smad3. The phosphorylated Smad3 recruits Smad4 and translocates into the cell nucleus. The Smad3/4 complex in the cell nucleus is able to activate EMT-inducers and lead to EMT and tumor metastasis. A recent study from Xue *et al.* showed that the mechanism of TGF-β-induced EMT in breast cancer cells is via the FOXM1 transactivation of Slug through the Smad pathway [55]. The activated Smad3/4 complex in the cell nucleus can also repress ID2 by interaction with transcription factor 3 (ATF3), thus enhancing Twist expression and leading to EMT [56]. Recently, it has also been reported that TGF-β can induce the CSC phenotype and cause EMT *in vitro* by up-regulation of Oct-4, Nanog, N-cadherin, Vimentin, Slug and Snail, and down-regulation of E-cadherin and Ck18. Knock-down of Oct-4 and Nanog inhibited the TGF-β-induced EMT [57]. The same study also showed that up-regulation of Oct-4 and Nanog in primary breast cancer tissue was associated with a poor prognosis. 

#### 6.1.2. Wnt Signaling

The Wnt/β-catenin signaling pathway is also implicated in playing a critical role in EMT in breast cancer cells. The Wnt signaling pathway can be initiated by the binding of Wnt ligands to transmembrane receptors of the Frizzled family. This induces stabilization of β-catenin by destruction of the APC complex, cell-cell adhesion, and E-cadherin. As a result, β-catenin accumulates in the nucleus, forming the β-catenin/TCF/LEF transcriptional complex, and drives transactivation of Wnt signaling–targeted genes which promote EMT [58,59]. Aberrations in the Wnt signaling pathway have been linked to many human cancers, including breast cancer, and appear to be associated with resistance to therapeutic treatment and aggressive types of cancer [60,61,62].

Data from our laboratory have shown that up-regulation of Wnt3 in trastuzumab-resistant HER2-overexpressing breast cancer cells activates the Wnt/β-catenin pathway and promotes the EMT-like transition [50]. In our study, knock-down of Wnt3 by siRNA restored cytoplasmic expression of β-catenin and decreased the EMT markers, suggesting that the EMT-like transition may be a unique feature of the cells acquiring resistance. Cell invasiveness was inhibited in response to Wnt3 down-regulation as well. Up-regulation of the Wnt signaling pathway was also found in MCF-7 cells when they acquired Tamoxifen resistance. Together, these data support the role of the Wnt signaling pathway in acquired resistance and link it to an EMT-like transition [62].

#### 6.1.3. Notch Signaling

In breast cancer patients, increased expression of Jagged1 (a ligand for the receptor Notch1) and Notch interactions has been shown to be predictive of poor overall survival [63]. *In* *vitro* studies have demonstrated that Notch participates in cancer metastasis by modulating EMT [64]. Jagged1-induced EMT has been shown as a mechanism which promotes tumor invasion and metastasis in breast cancer [65]. Data from the same study demonstrated that Jagged1-mediated Notch activation induces EMT through repression of E-cadherin by Slug. Notch-related EMT in breast cancer cells was also reported by Shao *et al.* [66]. Silencing Notch signaling showed growth arrest and inhibition of EMT in both breast cancer stem cells and breast cancer cells [67].

#### 6.1.4. Interaction of Signaling Pathways on EMT

Numerous evidence suggests that EMT can be regulated by the signaling cooperation and the convergence of different pathways on common targets [68]. Bakin *et al.* found that TGF-β-induced EMT was associated with the PI3K/Akt pathway. In their study, TGF-β induces EMT in mouse breast tumor line 4T1 via phosphorylation of Smad2. However, TGF-β-mediated phosphorylation of Smad2 was required to activate PI3K/Akt signaling. Co-incubation with PI3K inhibitor LY294002 reduced TGF-β-mediated Smad2 phosphorylation markedly, and at the same time phosphorylation of Akt at ser-473 was blocked completely [69].

In addition to the PIK3K pathway, TGF-β has been reported to activate the MAPK pathway and p38 MAPK-JNK pathway in relation to EMT [70,71,72,73]. Furthermore, TGF-β could induce ubiquitination of TRAF6 to activate TβRI and promote EMT via the p38 and JNK pathways [74,75]. Studies from our lab and others have also shown that activation of STAT3 can induce EMT [76]. Phosphorylation of STAT3 in MCF-7-HER2 cells (MCF-7 cells with HER2 expression) promoted CSC traits, including an increase in the number of CD44-positive cells. These cells also had increased Vimentin expression and decreased E-cadherin expression [76]. TGF-β can also facilitate EGF (Epidermal growth factor) or FGF (fibroblast growth factor)-inducing EMT [68,77,78]. Although both the TGF-β and Wnt/β-catenin signaling pathways play a dominant role in the initiation of EMT, the two pathways often cooperate in regulating gene expression and the EMT process [79].

It is apparent that gaining a stable mesenchymal phenotype requires sustained EMT-promoting signals, otherwise the metastatic mesenchymal phenotype may revert back to a more epithelial phenotype. As described in the mechanisms above, sustaining the EMT phenotype can be accomplished through autocrine and/or continuous paracrine signaling through the TGF-β and/or Wnt pathways [80]. Most recently, the epigenetic landscape has been proposed to govern the stability of EMT plasticity in tumor cells as well [81].

### 6.2. Epigenetics Regulation of EMT

Accumulating evidence indicates that epigenetic changes including DNA methylation, histone modification (methylation, acetylation, and ubiquitination), nucleosome remodeling, and alternations of microRNAs could regulate EMT in cancer cells [81,82,83,84].

#### 6.2.1. DNA Methylation and Histone Modification in Regulation of EMT

Hypermethylation of the E-cadherin promoter region has been frequently reported in human cancer including breast cancer [85,86,87]. The repression of E-cadherin by hypermethylation was confirmed by reversal of the repression by treatment with the demethylating agent 5′-aza-2′-deoxycytidine (5Aza-dC) in several cancer cell lines [85,86,87]. A hospital-based study conducted by Ulirsch *et al.* examined association of the DNA methylation of Vimentin in breast tumors and survival [88]. The status of Vimentin methylation was found to be inversely correlated with survival, but the sample size of the study was relatively small (n = 83).

Histone methylation can be associated with either transcriptional repression or activation. Changes in histone markers, such as H3K9me2 and H3K9me3, occur during the EMT process frequently. The epigenetic modification of histones can govern the stability of the EMT plasticity. Methylation of histone H3 on lysine 9 (H3K9me2) was reported to mediate E-cadherin transcriptional repression and drive tumor invasion and metastasis in endometrial cancer [89]. The histone H3 Lysine 9 methyltransferase, G9a, was also critical for Snail-mediated E-cadherin repression in human breast cancer [90,91].

Dong *et al*. found that knock-down of G9a suppressed H3K9me2 and restored E-cadherin expression [90]. Lysine-specific histone demethylase 1A (KDM1A), also known as lysine (K)-specific demethylase 1A (LSD1), can demethylate mono- and di-methylated lysines, specifically histone 3, and lysines 4 and 9 (H3K4 and H3K9). It has been reported that LSD1 is essential for Snai1-mediated transcriptional repression and for maintenance of the silenced state of Snai1 target genes in invasive cancer cells [91]. Data from their study showed that in the absence of LSD1, Snai1 failed to repress E-cadherin.

Furthermore, many of the EMT-TFs contain DNA binding domains and are able to mediate certain genes through the enhancer box (E-box) in gene promoters [92]. For instance, both Snail and Slug are known to bind to E-box elements and repress E-cadherin expression in breast cancer cells [93,94]. At the E-cadherin promoter, Snail binds to E-box elements and recruits HDAC1, HDAC2, and SIN3A complex to repress E-cadherin expression. Slug also binds to E-box elements, and recruits CtBP and HDAC3, thus also repressing E-cadherin and inducing EMT.

#### 6.2.2. MicroRNAs in Regulation of EMT

Recently, microRNAs (miRNAs), a new class of regulatory molecules, have been recognized as playing an important role in cancer metastases and they are regulated by epigenetic mechanisms [95,96]. Over 1400 miRNA have been identified in humans so far and de-regulation of miRNAs has profound consequences, such as inducing broad downstream changes in multiple genes [96]. Several miRNAs have been described as critical regulators in EMT, such as miR-200, miR-155, miR-137, miR-10b and miR-9 [97,98,99,100,101,102,103]. miR-9 and miR-115 have been reported to be elevated in invasive breast cancer tissues [98,102,103,104]. miR-9 promotes breast cancer progression through targeting CHD1, the E-cadherin-encoded gene [98]. miR-155 can mediate EMT due to its association with the TGF-β/Smad pathway [105,106], while the miR-200 family has been recognized as suppressors of EMT [107,108]. Eades *et al*. showed that miR-200 inhibits EMT through targeting ZEB1 and ZEB2, well-known transcriptional repressors of E-cadherin [107]. Inhibition or over-expression of miR-200c resulted in altered ZEB1 and ZEB2 expression and affected p53-regulated EMT in the normal mammary epithelial cell line MCF12A [108]. The same study also found that loss of p53 and decreased miR-200c in breast tumors were associated with increased expression of EMT markers and high tumor grade in breast tumors [108]. It has been suggested that p53 can regulate EMT through microRNAs targeting ZEB1 and ZEB2, as reported by Kim *et al*. [109]. In addition to miRNAs, unpublished data from our laboratory showed that long noncoding RNAs could also regulate EMT and tumor metastasis in breast cancer. MALAT1 (metastasis associated lung adenocarcinoma transcript (1)), known as NEAT2 (noncoding nuclear-enriched abundant transcript (2)), is a large, infrequently spliced non-coding RNA, which is highly conserved among mammals and highly expressed in the nucleus [110]. MALAT1 was originally identified as a prognostic marker for metastasis and patient survival in non-small-cell lung carcinoma (NSCLC) [111]. Up-regulation of MALAT1 has been associated with cell migration and tumor metastases in lung, bladder, cervical and colon cancers [111,112,113,114,115,116,117]. Abundant expression of MALAT1 was also identified in breast tumor sequences [118]. We have found that MALAT1 was significantly up-regulated in trastuzumab-resistant HER2 cells and in the more metastatic TNBC cell lines. The upregulation of MALAT1 in those cells was also associated with upregulation of EMT markers. Knock-down of MALAT1 by siRNA decreased EMT-TFs, Slug, Snail, and Twist significantly and inhibited cell invasion.

#### 6.2.3. Epigenetic Therapies and EMT

The reversibility of epigenetic alternations has attracted researchers and pharmaceutical companies to explore and develop novel targets for therapeutic intervention in cancer. Strategies include reversal of DNMTs (DNA methyltransferases) activity or inhibition of histone modification. Restoring E-cadherin expression and suppressing metastasis formation and primary tumor growth by 5-aza-2′-deoxycytidine has already been proven in the in vivo model of breast cancer using the MDA-MB435S cell line [119]. Currently, the majority of epigenetic drugs have been discovered to reverse DNA methylation and aberrant histone modifications, such as 5-aza-2′-deoxycytidine and HDAC inhibitors [120,121,122]. However, so far, clinical efficacy of those epigenetic therapies is still limited in solid tumors [123,124,125]. This is difficult for several reasons. Foremost, these broad de-methylating agents are associated with toxicities. Secondly, EMT is a highly dynamic process and involves multiple intermediate states—hence, targeting at the “right time” will be a challenge. The modulation of the epigenetic regulatory mechanisms for EMT needs to be further elucidated. An epigenetic therapeutic approach could prevent EMT occurrence, block metastasis, and, perhaps, also prevent cancer recurrence.

Chromatin-modifying enzymes are another class of molecules likely to play a significant role in the regulation of EMT. Recently, Vorinostat, a histone deacetylase inhibitor, has shown the ability to enhance the efficacy of carboplatin and paclitaxel in patients with advanced non-small-cell lung cancer (NSCLC) [126]. In this phase II randomized, double-blinded, placebo-controlled study, Ramalingam *et al*. evaluated the efficacy of Vorinostat in combination with carboplatin and paclitaxel in patients with advanced-stage NSCLC. Data from this study showed a trend toward improvement in median progression-free survival and overall survival in the Vorinostat arm. The miRNAs are also known to be able to regulate target genes and multiple cellular processes that involve the regulation of EMT. Hence, miRNA signaling has gained attention as a potential therapeutic target. Combination of epigenetic approaches and chemotherapies and/or cell signaling inhibitors could be a more promising way for the prevention of EMT and cancer metastasis.

## 7. Conclusions

As mentioned above, the majority of the evidence linking breast cancer progression with EMT is derived from studies in cancer cell lines and/or animal models. Therefore, its relevance in human cancer is still under debate and exploration. The reversibility of EMT-MET is one possible reason that has made this process difficult to definitively identify in human samples. Also, it has become increasingly evident that the EMT process may not simply result in cells with either the EMT or MET state. Rather, the EMT process involves a series of transitions and a spectrum of multiple intermediate states lying between the two cellular endpoints [80].

Validating biomarkers related to EMT in patient models will be highly crucial for identifying patients at risk of developing drug resistance and metastasis. A solid set of validated biomarkers could inform healthcare providers and patients in selecting treatment protocols and how aggressively they may choose to manage their cancers. The panel of markers may include CSC markers, EMT-TFs, key pathways regulators, non-coding RNAs, and mesenchymal or epithelial markers. Table 1 shows the clinical significance and prognosis value of EMT-related markers that have been evaluated using clinical samples. Clearly, the list of markers needs to be further validated and expanded. Furthermore, the method of detection of the biomarkers such as the antibodies used for immunohistochemistry needs to be validated and standardized as well, if they are to have broad clinical utility.

The advent of the human genome sequencing platform and gene profiling by microarray analysis has allowed us to stratify molecular signatures from tumor tissues. These will enable us to better identify the panel of biomarkers associated with the initiation of EMT and, possibly, assist in predicting the risk for tumor metastasis. The knowledge that we have gained from cell culture models with recent advances in imaging techniques will also enhance our ability to capture the EMT process during cancer progression in clinical samples. Understanding the biology of EMT and use of recent technologies and strategies will help in targeting EMT-associated cancer progression and designing customized therapies in the future. These strategies will give cancer patients hope to have the best selection of treatment protocols and improve disease outcome.

**Table 1:** Clinical significance of epithelial to mesenchymal transition (EMT)-related markers.

Study	N	Method	Markers	Clinical Outcome	*p*-Value
Lin *et al.* [26]	441	IHC	Low E-cadherin, High Slug, High Vimentin	Associated with Low DFS and Low OS	<0.01
Aleskandarany *et al.* [27]	1035	IHC, RPPA	Low E-cadhirin and High N-cadherin	Associated with Low DFS and Low OS	<0.001
Wu *et al.* [37]	126	IHC	CD44^+^/CD24^−^ *vs.* CD44^−^/CD24^−^;	Associated with Low DFS	0.05
			CD24^+/^CD44^−^ *vs.* CD44^−^/CD24^−^		0.016
Lin *et al.* [40]	147	IHC	CD44^high^/CD24^low^	Associated with Low DFS and Low OS	<0.05
Ma *et al.* [98]	45	RT-qPCR	miR9	Associated with Metastasis	<0.01
Bonnie *et al.* [127]	492	IHC	Low E-cadherin	Increased HR of all-cause mortality	<0.05
Khramtsov *et al.* [128]	117	IHC	High cytosolic β-catenin	Associated with Low OS	0.0005
			Or High or High nuclear β-catenin		0.039
Martin *et al.* [129]	190	RT-qPCR	High Twist	High mortality	n.s.
			High Snail	High mortality	n.s.
			High Slug	High metastasis	0.05
Mylona *et al.* [130]	155	IHC	CD44^+^/CD24^−^; CD24^+/^CD44^−^	No association with DFS and OS;	n.s.
				Associated with low DFS and OS	<0.05
Gwak *et al.* [131]	295	IHC, RT-qPCR	miR9	Associated with low DFS and OS	<0.05

IHC: Immunohistochemistry; RPPA: Reverse phase protein microarray; RT-qPCR: Reverse transcription real-time polymerase chain reaction; DFS: Five-year disease-free survival; OS: Overall survival; HR: hazard ratio; n.s.: no statistical significance; N: sample size.

## References

[1] Perou C.M., Sorlie T., Eisen M.B., van de Rijn M., Jeffrey S.S., Rees C.A., Pollack J.R., Ross D.T., Johnsen H., Akslen L.A. (2000). Molecular portraits of human breast tumors. Nature.

[2] Sorlie T., Perou C.M., Tibshirani R., Aas T., Geisler S., Johnsen H., Hastie T., Eisen M.B., van de Rijn M., Jeffrey S.S. (2001). Gene expression patterns of breast carcinomas distinguish tumor subclasses with clinical implications. Proc. Natl. Acad. Sci. USA.

[3] Sorlie T., Tibshirani R., Parker J., Hastie T., Marron J.S., Nobel A., Deng S., Johnsen H., Pesich R., Geisler S. (2003). Repeated observation of breast tumor subtypes in independent gene expression data sets. Proc. Natl. Acad. Sci. USA.

[4] Sotiriou C., Neo S.Y., McShane L.M., Korn E.L., Long P.M., Jazaeri A., Martiat P., Fox S.B., Harris A.L., Liu E.T. (2003). Breast cancer classification and prognosis based on gene expression profiles from a population-based study. Proc. Natl. Acad. Sci. USA.

[5] Sethi S., Sarkar F.H., Ahmed Q., Bandyopadhyay S., Nahleh Z.A., Semaan A., Sarkr W., Munkarah A., Ali-Fehmi R. (2011). Molecular markers of epithelial-to mesenchymal transition are associated with tumor aggressiveness in breast carcinoma. Translational. Oncol..

[6] Blick T., Widodo E., Hugo H., Waltham M., Lenburg M.E., Neve R.M., Thompson E.W. (2008). Epithelial mesenchymal transition traits in human breast cancer cell lines. Clin. Exp. Metastasis..

[7] Vogel C.L., Cobleigh M.A., Tripathy D., Gutheil J.C., Harris L.N., Fehrenbacher L., Slamon D.J., Murphy M., Novotny W.F., Burchmore M. (2002). Efficacy and safety of trastuzumab as a single agent in first-line treatment of HER2-overexpressing metastatic breast cancer. J. Clin. Oncol..

[8] Montagna E., Cancello G., D’Agostino D., Lauria R., Forestieri V., Esposito A., Silvestro L., Accurso A., de Placido S., de Laurentiis M. (2009). Central nervous system metastases in a cohort of metastatic breast cancer patients treated with trastuzumab. Cancer Chemother. Pharmacol..

[9] Clayton A.J., Danson S., Jolly S., Ryder W.D.J., Burt P.A., Stewart A.L., Wilkinson P.M., Welch R.S., Magee B., Wilson G. (2004). Incidence of cerebral metastases in patients treated with trastuzumab for metastatic breast cancer. Br. J. Cancer.

[10] Thiery J.P., Lim C.T. (2013). Tumor dissemination: An EMT affair. Cancer Cell.

[11] Christofori G., Bill R. (2015). The relevance of EMT in breast cancer metastasis: Correlation or causality. FEBS.

[12] Fidler I.J., Kripke M.L. (1977). Metastasis results from preexisting variant cells within a malignant tumor. Science.

[13] Daleba P., Cho R.W., Clarke M.F. (2007). Cancer stem cells: Models and concepts. Annu. Rev. Med..

[14] Mani S.A., Guo W., Liao M.J., Eaton E.N., Ayyanan A., Zhou A.Y., Brooks M., Reinhard F., Zhang C.C., Shipitsin M. (2008). The epithelial-mesenchymal transition generates cells with properties of stem cells. Cell.

[15] Blick T., Hugo H., Widodo E., Waltham M., Pinto C., Mani S.A., Weinberg R.A., Neve R.M., Lenburg M.E., Thompson E.W. (2010). Epithelial mesenchymal transition traits in human breast cancer cell lines parallel the CD44^hi/^CD24^lo/−^ stemcell phenotype in human breast cancer. J. Mammary Gland Biol. Neoplasia..

[16] Iwatsuki M., Mimori K., Yokobori T., Ishi H., Beppu T., Nakamori S., Baba H., Mori M. (2010). Epithelia-mesenchymal transition in cancer development and its clinical significance. Cancer Sci..

[17] Thiery J.P., Acloque H., Huang R.Y.J., Nieto M.A. (2009). Epithelial-mesenchymal transitions in development and disease. Cell.

[18] Foroni C., Broggini M., Generali D., Damia G. (2012). Epithelial-mesenchymal transition and breast cancer: Role, molecular mechanisms and clinical impact. Cancer Treat. Rev..

[19] Mallini P., Lennard T., Kriby J., Meeson A. (2014). Epithelial-to-mesenchymal transition: What is the impact on breast cancer stem cells and drug resistance. Cancer Treat. Rev..

[20] Lien H.C., Hsiao Y.H., Lin Y.S., Yao Y.T., Juan H.F., Kuo W.H., Hung M.C., Chang K.J., Hsieh F.J. (2007). Molecular signatures of metaplastic carcinoma of the breast by large-scale transcriptional profiling: Identification of genes potentially related to epithelial-mesenchymal transition. Oncogene.

[21] Bebee T.W., Cieply B.W., Carstens R.P. (2014). Genome-wide activities of RNA binding proteins that regulate cellular changes in the epithelial to mesenchymal transition (EMT). Adv. Exp. Med. Biol..

[22] Cheng Q., Chang J.T., Gwin W.R., Zhu J., Ambs S., Geradts J., Lyerly H.K. (2014). A signature of epithelial-mesenchymal plasticity and stromal activation in primary tumor modulates late recurrence in breast cancer independent of disease subtype. Breast Cancer Res..

[23] Hazan R.B., Qiao R., Keren R., Badano I., Suyama K. (2004). Cadherin switch in tumor progression. Ann. N. Y. Acad. Sci..

[24] Maeda M., Johnson K.R., Wheelock M.J. (2005). Cadherin switching: Essential for behavioral but not morphological changes during an epithelium-to-mesenchyme transition. J. Cell Sci..

[25] Sarrió D., Rodriguez-Pinilla S.M., Hardisson D., Cano A., Moreno-Bueno G., Palacios J. (2008). Epithelial-mesenchymal transition in breast cancer relates to the basal-like phenotype. Cancer Res..

[26] Liu T., Zhang X., Shang M., Zhang Y., Xia B., Niu M., Liu Y., Pang D. (2013). Dysregulated expression of Slug, Vimentin, and E-cadherin correlates with poor clinical outcome in patients with basal-like breast cancer. J. Surg. Oncol..

[27] Aleskandarany M.A., Negm O.H., Green A.R., Ahmed M.A.H., Nolan C.C., Tighe P.J., Ellis I., Rakha E.A. (2014). Epithelial mesenchymal transition in early invasive breast cancer: An immunohistochemical and reverse phase protein array study. Breast Cancer Res. Treat..

[28] Choi Y., Lee H.J., Jang M.H., Gwak J.M., Lee K.S., Kim E.J., Kim H.J., Lee H.E., Park S.Y. (2013). Epithelial-mesenchymal transition increases during the progression of *in situ* to invasive basal-like breast cancer. Hum. Pathol..

[29] Tsang J.Y., Au S.K., Ni Y.B., Shao M.M., Siu W.M., Hui S.W., Chan S.K., Chan K.W., Kwok Y.K., Chan K.F. (2013). P-cadherin and Vimentin are useful basal markers in breast cancers. Hum. Pathol..

[30] Yu M., Bardia A., Wittner B.S., Stott S.L., Smas M.E., Ting D.T., Isakoff S.J., Ciciliano J.C., Wells M.N., Shah A.M. (2013). Circulating breast tumor cells exhibit dynamic changes in epithelial and mesenchymal composition. Science.

[31] Armstrong A.J., Marengo M.S., Oltean S., Kemeny G., Bitting R.L., Turnbull J.D., Herold C.I., Marcom P.K., George D.J., Garcia-Blanco M.A. (2011). Circulating tumor cells from patients with advanced prostate and breast cancer display both epithelial and mesenchymal markers. Mol. Cancer Res..

[32] Papadaki M.A., Kallergi G., Zafeiriou Z., Manouras L., Theodoropoulos P.A., Mavroudis D., Georgoulias V., Agelaki S. (2014). Co-expression of putative stemness and epithelial-to-mesenchymal transition markers on single circulating tumour cells from patients with early and metastatic breast cancer. BMC Cancer.

[33] Kallergi G., Papadaki M.A., Politaki E., Mavroudis D., Georgoulias V., Agelaki S. (2011). Epithelial to mesenchymal transition markers expressed in circulating tumour cells of early and metastatic breast cancer patients. Breast Cancer Res..

[34] Radisky D.C., LaBarge M.A. (2008). Epithelial-mesenchymal transition and the stem cell phenotype. Cell Stem Cell.

[35] AL-Hajj M., Wicha M.S., Benito-Hernandez A., Morrison S.J., Clarke M.F. (2003). Prospective identification of tumorigenic breast cancer cells. PNAS.

[36] Liu S., Cong Y., Wang D., Sun Y., Deng L., Liu Y., Martin-Trevino R., Shang L., McDermott S.P., Landis M.D. (2013). Breast cancer stem cells transition between epithelial and mesenchymal states reflective of their normal counterparts. Stem Cell Rep..

[37] Wu Y., Sarkissyan M., Elshimali Y., Vadgama J.V. (2013). Triple negative breast tumors in African-American and Hispanic/Latina women are high in CD44^+^, low in CD24^+^, and have loss of PTEN. PLoS ONE.

[38] Honeth G., Bendahl P.O., Ringnér M., Saal L.H., Gruvberger-Saal S.K., Lövgren K., Grabau D., Fernö M., Borg A. (2008). The CD44+/CD24− phenotype is enriched in basal-like breast tumors. Breast Cancer Res..

[39] Giatromanolaki A., Sivridis E., Fiska A., Koukourakis M.I. (2011). The CD44^+^/CD24^−^ phenotype relates to “triple-negative” state and unfavorable prognosis in breast cancer patients. Med. Oncol..

[40] Lin Y., Zhong Y., Guan H., Zhang X., Sun Q. (2012). CD44^+^/CD24^−^ phenotype contributes to malignant relapse following surgical resection and chemotherapy in patients with invasive ductal carcinoma. J. Exp. Clin. Cancer Res..

[41] Perrone G., Gaeta L.M., Zagami M., Nasorri F., Coppola R., Borzomati D., Bartolozzi F., Altomare V., Trodella L., Tonini G. (2012). *In situ* identification of CD44^+^/CD24^−^ cancer cells in primary human breast carcinomas. PLOS ONE.

[42] Fischer K.R., Durrans A., Lee S., Sheng J., Li F., Wong S.T., Choi H., El Rayes T., Ryu S., Troeger J. (2015). Epithelial-to-mesenchymal transition is not required for lung metastasis but contributes ro chemoresistance. Nature.

[43] Li Q., Xu J., Wang W., Cao X., Chen Q., Tang F., Chen Z.Q., Liu X.P., Xu Z.D. (2009). Twist1-mediated Adriamycin-induced epithelial-mesenchymal transition relates to multidrug resistance and invasive potential in breast cancer cells. Clin. Cancer Res..

[44] Yang Q., Huang J., Wu Q., Cai Y., Zhu L., Lu X., Chen S., Chen C., Wang Z. (2014). Acquisition of epithelial-mesenchymal transition is associated with Skp2 expression in paclitaxel-resistant breast cancer cells. Br. J. Cancer.

[45] Zhang W., Feng M., Zheng G., Chen Y., Wang X., Pen B., Yin J., Yu Y., He Z. (2012). Chemoresistance to 5-fluorouracil induces epithelial-mesenchymal transition via up-regulation of Snail in MCF7 human breast cancer cells. Biochem. Biophys. Res. Commun..

[46] Işeri Ö.D., Kars M.D., Arpaci F., Atalay C., Pak I., Gündüz U. (2011). Drug resistant MCF-7 cells exhibit epithelial-mesenchymal transition gene expression pattern. Biomed. Phamacother..

[47] Hiscox S., Jiang W.G., Obermeier K., Taylor K., Morgan L., Burmi R., Barrow D., Nicholson R.I. (2006). Tamoxifen resistance in MCF7 cells promotes EMT-like behavior and involves modulation of β-catenin phosphorylation. Int. J. Cancer.

[48] Liu H., Zhang H.W., Sun X.F., Guo X.H., He Y.N., Cui S.D., Fan Q.X. (2013). Tamoxifen-resistant breast cancer cells possess cancer stem-like cell properties. Chin. Med. J..

[49] Oliveras-Ferraros C., Corominas-Faja B., Cufi S., Vazquez-Martin A., Martin-Castillo B., Iglesias J.M., López-Bonet E., Martin Á.G., Menendez J.A. (2012). Epithelial-to mesenchymal transition (EMT) confers primary resistance to trastuzumab (Herceptin). Cell Cycle.

[50] Wu Y., Ginther C., Kim J., Mosher N., Chung S., Slamon D., Vadgama J.V. (2012). Expression of Wnt3 activates Wnt/β-catenin pathway and promotes EMT-like phenotype in trastuzumab-resistant HER2-overexpressing breast cancer cells. Mol. Cancer Res..

[51] Mego M., Mani S.A., Lee B.N., Li C., Evans K.W., Cohen E.N., Gao H., Jackson S.A., Giordano A., Hortobagyi G.N. (2012). Expression of epithelial-mesenchymal transition-inducing transcription factors in primary breast cancer: The effect of neoadjuvant therapy. Int. J. Cancer.

[52] Feng X., Derynck R. (2005). Specificity and versatility in TGF-β signaling though Smads. Annu. Rev. Cell Dev. Biol..

[53] Massagué J. (2012). TGFβ signaling in context. Nat. Rev. Mol. Cell Biol..

[54] Heldin C., Moustakas A. (2012). Role of Smads in TGFβ signaling. Cell Tissue Res..

[55] Xue J., Lin X., Chiu W.T., Chen Y.H., Yu G., Liu M., Feng X.H., Sawaya R., Medema R.H., Hung M.C. (2014). Sustained activation of SMAD3/SMAD4 by FOXM1 promotes TGF-β-dependent cancer metastasis. J. Clin. Invest..

[56] Kang Y., Chen C.R., Massagué J. (2003). A self-enabling TGFβ response coupled to stress signaling: Smad engages stress response factor ATF3 for Id1 repression in epithelial cells. Mol. Cell.

[57] Wang D., Lu P., Zhang H., Luo M., Zhang X., Wei X., Gao J., Zhao Z., Liu C. (2014). Oct-4 and Nanog promote the epithelial-mesenchymal transition of breast cancer stem cells and are associated with poor prognosis in breast cancer patients. Oncotarget.

[58] Yook J.I., Li X.Y., Ota I., Hu C., Kim H.S., Kim N.H., Cha S.Y., Ryu J.K., Choi Y.J., Kim J. (2006). A Wnt-Axin2-GSK3β cascade regulates Snail1 activity in breast cancer cells. Nat. Cell Biol..

[59] Gilles C., Polette M., Mestdagt M., Nawrocki-Raby B., Ruggeri P., Birembaut P., Foidart J.M. (2003). Transactivation of Vimentin by β-catenin in human breast cancer cells. Cancer Res..

[60] Li Y., Ma C., Shi X., Wen Z., Li D., Sun M., Ding H. (2014). Effect of nitric oxide synthase on multiple drug resistance is related to Wnt signaling in non-small cell lung cancer. Oncol. Rep..

[61] Gheidari F., Bakhshandeh B., Teimoori-Toolabi L., Mehrtash A., Ghadir M., Zeinali S. (2014). TCF4 silencing sensitizes the colon cancer cell line to oxaliplatin as a common chemotherapeutic drug. Anticancer Drugs.

[62] Loh Y.N., Hedditch E.L., Baker L.A., Jary E., Ward R.L., Ford C.E. (2013). The Wnt signaling pathway is upregulated in an *in vitro* model of acquired tamoxifen resistant breast cancer. BMC Cancer.

[63] Reedijk M., Odorcic S., Chang L., Zhang H., Miller N., McCready D.R., Lockwood G., Egan S.E. (2005). High-level coexpression of JAG1 and NOTCH1 is observed in human breast cancer and is associated with poor overall survival. Cancer Res..

[64] Wang Z., Li Y., Kong D., Sarkar F.H. (2010). The Role of Notch signaling pathway in epithelial-mesenchymal transition (EMT) during development and tumor aggressiveness. Curr. Drug Targets.

[65] Leong K.G., Niessen K., Kulic I., Raouf A., Eaves C., Pollet I., Karsan A. (2007). Jagged1-mediated Notch activation induces epithelial-to-mesenchymal transition through Slug-induced repression of E-cadherin. J. Exp. Med..

[66] Shao S., Zhao X., Zhang X., Luo M., Zuo X., Huang S., Wang Y., Gu S., Zhao X. (2015). Notch1 signaling regulates the epithelial-mesenchymal transition and invasion of breast cancer in a Slug-dependent manner. Mol. Cancer.

[67] Suman S., Das T.P., Damodaran C. (2013). Silencing NOTCH signaling causes growth arrest in both breast cancer stem cells and breast cancer cells. Br. J. Cancer.

[68] Lamouille S., Xu J., Derynck R. (2014). Molecular mechanisms of epithelial-mesenchymal transition. Nat. Rev..

[69] Bakin A.V., Tomlinson A.K., Bhowmick N.A., Moses H.L., Arteaga C.L. (2000). Phosphatidylinositol 3-kinase function is required for transforming growth factor β-mediated epithelial to mesenchymal transition and cell migration. J. Bio. Chem..

[70] Lamouille S., Derynck R. (2011). Emergence of the phosphoinositide 3-kinase-Akt-mammalian target of rapamycin axis in transforming growth factor-β-induced epithelial-mesenchymal transition. Cells Tissues Organs.

[71] Lamouille S., Derynck R. (2007). Cell size and invasion in TGF-β—Induced epithelial to mesenchymal transition is regulated by activation of the mTOR pathway. J. Cell Biol..

[72] Lamouille1 S., Connolly E., Smyth J.W., Akhurst R.J., Derynck R. (2012). TGF-β-induced activation of mTOR complex 2 drives epithelial-mesenchymal transition and cell invasion. J. Cell Sci..

[73] Xie L., Law B.K., Chytil A.M., Brown K.A., Aakre M.E., Moses H.L. (2004). Activation of the Erk pathway is required for TGF-β-induced EMT *in vitro*. Neoplasia.

[74] Yamashita M., Fatyol K., Jin C., Wang X., Liu Z., Zhang Y.E. (2008). TRAF6 mediates Smad-independent activation of JNK and p38 by TGF-β. Mol. Cell.

[75] Sorrentino A., Thakur N., Grimsby S., Marcusson A., von Bulow V., Schuster N., Zhang S., Heldin C.H., Landström M. (2008). The type I TGF-β receptor engages TRAF6 to activate TAK1 in a receptor kinase-independent manner. Nat. Cell Biol..

[76] Chung S., Giehl N., Wu Y., Vadgama J.V. (2014). STAT3 activation in HER2-overexpressing breast cancer promotes epithelial-mesenchymal transition and cancer stem cell traits. Int. J. Oncol..

[77] Wendt M.K., Smith J.A., Schiemann W.P. (2010). Transforming growth factor-β-induced epithelial-mesenchymal transition facilitates epidermal growth factor-dependent breast cancer progression. Oncogene.

[78] Shirakihara T., Horiguchi K., Miyazawa K., Ehata S., Shibata T., Morita I., Miyazono K., Saitoh M. (2011). TGF-β regulates isoform switching of FGF receptors and epithelial-mesenchymal transition. EMBO J..

[79] Medici D., Hay E.D., Goodenough D.A. (2006). Cooperation between snail and LEF-1 transcription factors is essential for TGF-β1-induced epithelial-mesenchymal transition. Mol. Biol Cell.

[80] Acloque H., Adams M.S., Fishwick K., Bronner-Fraser M., Nieto M.A. (2009). Epithelial-mesenchymal transitions: the importance of changing cell state in development and disease. J. Clin. Invest..

[81] Tam W.L., Weinberg R.A. (2013). The epigenetics of epithelial-mesenchymal plasticity in cancer. Nat. Med..

[82] Nickel A., Stadler S.C. (2015). Role of epigenetic mechanisms in epithelial-to-mesenchymal transition of breast cancer cells. Transl. Res..

[83] Lujambio A., Esteller M. (2009). How epigenetics can explain human metastasis: A new role for microRNAs. Cell Cycle.

[84] Wang Y., Shang Y. (2013). Epigenetic control of epithelial-to-mesenchymal transition and cancer metastasis. Exp. Cell Res..

[85] Graff J.R., Herman J.G., Lapidus R.G., Chopra H., Xu R., Jarrard D.F., Isaacs W.B., Pitha P.M., Davidson N.E., Baylin S.B. (1995). E-cadherin expression is silenced by DNA hypermethylation in human breast and prostate carcinomas. Cancer Res..

[86] Yoshiura K., Kanai Y., Ochiai A., Shimoyama Y., Sugimura T., Hirohashi S. (1995). Silencing of the E-cadherin invasion-suppressor gene by CpG methylation in human carcinomas. Proc. Natl. Acad. Sci. USA.

[87] Graff J.R., Gabrielson E., Fujii H., Baylin S.B., Herman J.G. (2000). Methylation patterns of the E*-cadherin* 5′-CpG island are unstable and reflect the dynamic, heterogeneous loss of E-cadherin expression during metastatic progression. J. Biol. Chem..

[88] Ulirsch J., Fan C., Knafl G., Wu M.J., Coleman B., Perou C.M., Swift-Scanlan T. (2013). Vimentin DNA methylation predicts survival in breast cancer. Breast Cancer Res. Treat..

[89] Hsiao S.M., Chen M.W., Chen C.A., Chien M.H., Hua K.T., Hsiao M., Kuo M.L., Wei L.H. (2015). The H3K9 Methyltransferase G9a Represses E-cadherin and is Associated with Myometrial Invasion in Endometrial Cancer. Ann. Surg. Oncol..

[90] Dong C., Wu Y., Yao J., Wang Y., Yu Y., Rychahou P.G., Evers B.M., Zhou B.P. (2012). G9a interacts with Snail and is critical for Snail-mediated E-cadherin repression in human breast cancer. J. Clin. Invest..

[91] Lin T., Ponn A., Hu X., Law B.K., Lu J. (2010). Requirement of the histone demethylase LSD1 in Snai1-mediated transcriptional repression during epithelial-mesenchymal transition. Oncogene.

[92] Peinado H., Olmeda D., Cano A. (2007). Snail, Zeb and bHLH factors in tumour progression: An alliance against the epithelial phenotype?. Nat. Rev. Cancer.

[93] Bolós V., Peinado H., Pérez-Moreno M.A., Fraga M.F., Esteller M., Cano A. (2003). The transcription factor Slug represses E-cadherin expression and induces epithelial to mesenchymal transitions: A comparison with Snail and E47 repressors. J. Cell Sci..

[94] Hajra K.M., Chen D.Y., Fearon E.R. (2002). The SLUG zinc-finger protein represses E-cadherin in breast cancer. Cancer Res..

[95] Bartel D.P. (2009). MicroRNAs: Target Recognition and Regulatory Functions. Cell.

[96] Bullock M.D., Sayan A.E., Packham G.K., Mirnezami A.H. (2012). MicroRNAs: Critical regulators of epithelial to mesenchymal (EMT) and mesenchymal to epithelial transition (MET) in cancer progression. Biol. Cell.

[97] Gibbons D.L., Lin W., Creighton C.J., Rizvi Z.H., Gregory P.A., Goodall G.J., Thilaganathan N., Du L., Zhang Y., Pertsemlidis A. (2009). Contextual extracellular cues promote tumor cell EMT and metastasis by regulating miR-200 family expression. Genes Dev..

[98] Ma L., Young J., Prabhala H., Pan E., Mestdagh P., Muth D., Teruya-Feldstein J., Reinhardt F., Onder T.T., Valastyan S. (2010). miR-9, a MYC/MYCN-activated microRNA, regulates E-cadherin and cancer metastasis. Nat. Cell Biol..

[99] Ma L., Teruya-Feldstein J., Weinberg R.A. (2007). Tumor invasion and metastasis initiated by microRNA-10b in breast cancer. Nature.

[100] Deng Y., Deng H., Bi F., Liu J., Bemis L.T., Norris D., Wang X.J., Zhang Q. (2011). MicroRNA-137 targets carboxyl-terminal binding protein 1 in melanoma cell lines. Int. J. Biol. Sci..

[101] Brabletz S., Brabletz T. (2010). The ZEB/miR-200 feedback loop—A motor of cellular plasticity in development and cancer?. EMBO Rep..

[102] Yu D.D., Lv M.M., Chen W.X., Zhong S.L., Zhang X.H., Chen L., Ma T.F., Tang J.H., Zhao J.H. (2015). Role of miR-155 in drug resistance of breast cancer. Tumor Biol..

[103] Mattiske S., Suetani R.J., Neilsen P.M., Callen D.F. (2012). The oncogenic role of miR-155 in breast cancer. Cancer Epidemiol. Biomarkers Prev..

[104] Neilsen P.M., Noll J.E., Mattiske S., Bracken C.P., Gregory P.A., Schulz R.B., Lim S.P., Kumar R., Suetani R.J., Goodall G.J. (2013). Mutant p53 drives invasion in breast tumors through up-regulation of miR-155. Oncogene.

[105] Kong W., Yang H., He L., Zhao J.J., Coppola D., Dalton W.S., Cheng J.Q. (2008). MicroRNA-155 is regulated by the transforming growth factor β/Smad pathway and contributes to epithelial cell plasticity by targeting RhoA. Mol. Cell Biol..

[106] Johansson J., Berg T., Kurzejamska E., Pang M.F., Tabor V., Jansson M., Roswall P., Pietras K., Sund M., Religa P. (2013). MiR-155-mediated loss of C/EBPβ shifts the TGF-β response from growth inhibition to epithelial-mesenchymal transition, invasion and metastasis in breast cancer. Oncogene.

[107] Eades G., Yao Y., Yang M., Zhang Y., Chumsri S., Zhou Q. (2011). miR-200a regulates SIRT1 expression and epithelial to mesenchymal transition (EMT)-like transformation in mammary epithelial cells. J. Biol. Chem..

[108] Chang C.J., Chao C.H., Xia W., Yang J.Y., Xiong Y., Li C.W., Yu W.H., Rehman S.K., Hsu J.L., Lee H.H. (2011). p53 regulates epithelial-mesenchymal transition and stem cell properties through modulating miRNAs. Nat. Cell Biol..

[109] Kim T., Veronese A., Pichiorri F., Lee T.J., Jeon Y.J., Volinia S., Pineau P., Marchio A., Palatini J., Suh S.S. (2011). p53 regulates epithelial-mesenchymal transition through microRNAs targeting ZEB1 and ZEB2. J. Exp. Med..

[110] Hutchinson J.N., Ensminger A.W., Clemson C.M., Lynch C.R., Lawrence J.B., Chess A. (2007). A screen for nuclear transcripts identifies two linked noncoding RNAs associated with SC35 splicing domains. BMC Genomics.

[111] Ji P., Diederichs S., Wang W., Böing S., Metzger R., Schneider P.M., Tidow N., Brandt B., Buerger H., Bulk E. (2003). MALAT1, a novel noncoding RNA, and thymosin β4 predict metastasis and survival in early-stage non-small cell lung cancer. Oncogene.

[112] Schmidt L.H., Spieker T., Koschmieder S., Schäffers S., Humberg J., Jungen D., Bulk E., Hascher A., Wittmer D., Marra A. (2011). The long noncoding MALAT1 RNA indicates a poor prognosis in non-small cell lung cancer and induces migration and tumor growth. J. Thorac. Oncol..

[113] Gutschner T., Hammerle M., Eissmann M., Hsu J., Kim Y., Hung G., Revenko A., Arun G., Stentrup M., Gross M. (2013). The noncoding RNA MALAT1 is a critical regulator of the metastasis phenotype of lung cancer cells. Cancer Res..

[114] Han Y., Liu Y., Nie L., Gui Y., Cai Z. (2013). Inducing cell proliferation inhibition, apoptosis, and motility reduction by silencing long noncoding ribonucleic acid metastasis-associated lung adenocarcinoma transcript 1 in urothelial carcinoma of the bladder. Urology.

[115] Ying L., Chen Q., Wang Y., Zhou Z., Huang Y., Qiu F. (2012). Upregulated MALAT1 contributes to bladder cancer cell migration by inducing epithelial-to-mesenchymal transition. Mol. Biosyst..

[116] Xu C., Yang M., Tian J., Wang X., Li Z. (2011). MALAT1: A long non-coding RNA and its important 3′ end functional motif in colorectal cancer metastasis. Int. J. Oncol..

[117] Gutschner T., Hämmerle M., Diederichs S. (2013). MALAT1—A paradigm for long noncoding RNA function in cancer. J. Mol. Med..

[118] Guffanti A., Iacono M., Pelucchi P., Kim N., Solda G., Croft L.J., Taft R.J., Rizzi E., Askarian-Amiri M., Bonnal R.J. (2009). A transcriptional sketch of a primary human breast cancer by 454 deep sequencing. BMC Genomics.

[119] Nam J.S., Ino Y., Kanai Y., Sakamoto M., Hirohashi S. (2004). 5-aza-2′-deoxycytidine restores the E-cadherin system in E-cadherin-silenced cancer cells and reduces cancer metastasis. Clin. Exp. Metastasis..

[120] Issa J.P., Kantarjian H.M. (2009). Targeting DNA methylation. Clin. Cancer Res..

[121] Lane A.A., Chabner B.A. (2009). Histone deacetylase inhibitors in cancer therapy. J. Clin. Oncol..

[122] Kelly T.K., de Carvalho D.D., Jones P.A. (2010). Epigenetic modifications as therapeutic targets. Nat. Biotechnol..

[123] Braiteh F., Soriano A.O., Garcia-Manero G., Hong D., Johnson M.M., de Padua Silva L., Yang H., Alexander S., Wolff J., Kurzrock R. (2008). Phase I study of epigenetic modulation with 5-azacytidine and valproic acid in patients with advanced cancers. Clin. Cancer Res..

[124] Lin J., Gilbert J., Rudek M.A., Zwiebel J.A., Gore S., Jiemjit A., Zhao M., Baker S.D., Ambinder R.F., Herman J.G. (2009). A phase I dose-finding study of 5-azacytidine in combination with sodium phenylbutyrate in patients with refractory solid tumors. Clin. Cancer Res..

[125] Munster P.N., Marchion D., Thomas S., Egorin M., Minton S. (2009). Phase I trial of vorinostat and doxorubicin in solid tumours: Histone deacetylase 2 expression as a predictive marker. Br. J. Cancer..

[126] Ramalingam S.S., Maitland M.L., Frankel P., Argiris A.E., Koczywas M., Gitlitz B., Thomas S., Espinoza-Delgado I., Vokes E.E., Gandara D.R. (2010). Carboplatin and paclitaxel in combination with either vorinostat or placebo for first-line therapy of advanced non-small-cell lung cancer. J. Clin. Oncol..

[127] Bonnie E., Rothberg G., Bracken M.B. (2006). E-cadherin immunohistochemical expression as a prognostic factor in infiltrating ductal carcinoma of the breast: A systematic review and meta-analysis. Breast Cancer Res. Treat..

[128] Khramtsov A.I., Khramtsova G.F., Tretiakova M., Huo D., Olopade O.I., Goss K.H. (2010). Wnt/β-catenin pathway activation is enriched in basal-like breast cancers and predicts poor outcome. Am. J. Pathol..

[129] Martin T.A., Goyalm A., ChB M.B., Watkins G., Kiang W.G. (2005). Expression of the transcription factors Snail, Slug, and Twist and their clinical significance in human breast cancer. Ann. Surg. Oncol..

[130] Mylona E., Giannopoulou I., Fasomytakis E., Nomikos A., Magkou C., Bakarakos P., Nakopoulou L. (2008). The clinicopathologic and prognostic significance of CD44^+^/CD24^−/low^ and CD44^−^/CD24^+^ tumor cells in invasive breast carcinomas. Hum. Pathol..

[131] Gwak J.M., Kim H.J., Kim E.J., Chung Y.R., Yun S., Seo A.N., Lee H.J., Park S.Y. (2014). MicroRNA-9 is associated with epithelial-mesenchymal transition, breast cancer stem cell phenotype, and tumor progression in breast cancer. Breast Cancer Res. Treat..

